# Trends in incidence and mortality for ovarian cancer in China from 1990 to 2019 and its forecasted levels in 30 years

**DOI:** 10.1186/s13048-023-01233-y

**Published:** 2023-07-14

**Authors:** Jianyang Feng, Lijiang Xu, Yangping Chen, Rongjin Lin, Haoxian Li, Hong He

**Affiliations:** 1 Department of Obstetrics and Gynecology, Guangdong Provincial Key Laboratory of Major Obstetric Disease, The Third Affiliated Hospital of Guangzhou Medical University, Guangzhou, 510000 China; 2 Department of Obstetrics and Gynecology, The First People’s Hospital of Foshan, Foshan, 528000 China

**Keywords:** Ovarian cancer, Incidence, Mortality, Disease burden, Projection, China

## Abstract

**Background:**

The specific long-term trend in ovarian cancer (OC) rates in China has been rarely investigated. We aimed to estimate the temporal trends in incidence and mortality rates from 1990 to 2019 in OC and predict the next 30-year levels. Data on the incidence, mortality rates, and the number of new cases and deaths cases due to OC in the China cohort from 1990 to 2019 were retrieved from the Global Burden of Disease Study 2019. Temporal trends in incidence and mortality rates were evaluated by joinpoint regression models. The incidence and mortality rates and the estimated number of cases from 2020 to 2049 were predicted using the Bayesian age–period–cohort model.

**Results:**

Consecutive increasing trends in age-standardized incidence (average annual percent change [AAPC] = 2.03; 95% confidence interval [CI], 1.90–2.16;* p* < 0.001) and mortality (AAPC = 1.58; 95% CI, 1.38−1.78; *p* < 0.001) rates in OC were observed from 1990–2019 in China. Theoretically, both the estimated age-standardized (per 100,000 women) incidence (from 4.77 in 2019 to 8.95 in 2049) and mortality (from 2.88 in 2019 to 4.03 in 2049) rates will continue to increase substantially in the coming 30 years. And the estimated number of new cases of, and deaths from OC will increase by more than 3 times between 2019 and 2049.

**Conclusions:**

The disease burden of OC in incidence and mortality has been increasing in China over the past 30 years and will be predicted to increase continuously in the coming three decades.

**Supplementary Information:**

The online version contains supplementary material available at 10.1186/s13048-023-01233-y.

## Background

Ovarian cancer (OC) comprises several histopathologic phenotypes that primarily include epithelial, sex cord-stromal, and germ cell cancers, which is the most lethal gynecological cancer with an estimated 57,200 new cases and 27,200 deaths in 2016 in China [1]. Unfortunately, there are no effective screening methods to early detect OC heretofore. And the majority of OC (the bulk of which are epithelial carcinomas) are advanced stage at diagnosis with a high recurrent rate and dismal prognosis. Accompanied by an aging population and the ongoing process of China’s reform and opening, both the incidence and mortality trends for OC in China have changed over time. Recently, Wang et al. investigated the mortality trends of OC in Chinese women and found that OC has exceeded uterine cancer as the second leading cause of death after 2005 with significant age, cohort, and period effects [2]. However, they neither estimated the annual percent change (APC) and average APC (AAPC)—measures to summarize the trend over a fixed predetermined interval, nor the incidence trends of OC in Chinese women. In our study, we estimated the APC and AAPC in incidence and mortality of OC in China from 1990 to 2019 and predicted the disease burden trends for the coming 30 years via the Bayesian age–period–cohort model, which may facilitate the governors to establish relevant health policies that can guide practices to reduce the burden of ovarian cancer in China.

## Methods

### Data resource

The China National Central Cancer Registry (CNCCR) takes the responsibility to collect, register and count all cancer-related data. The Global Health Data Exchange (GHDx) is an international open-source platform to estimate the Global Burden of Disease (GBD) worldwide. And CNCCR is one of the contributors to GHDx. The latest version–GBD 2019 results has been released. This nationwide population-based study data of the Chinese OC cohort including the annual number of incidence and mortality cases and standardized rates were collected via the GHDx query tool (available at http://ghdx.healthdata.org/gbd-results-tool) from 1990 to 2019. The estimated female population of China by 5-year age groups was downloaded from the United Nations World Population Prospects 2019 (available at https://population.un.org/wpp/Download/Standard/CSV/). The present study did not involve any human subjects or personal identity information. The Ethics Approval application was not requisite.

### Statistical analysis

The APC and AAPC were evaluated by joinpoint regression analysis (Joinpoint Regression Program, Version 4.9.0.1., Statistical Research and Applications Branch, National Cancer Institute, MD, USA) to determine the temporal incidence and mortality trends of OC in China from 1900 to 2019. A logarithmic transformation of the measured standardized rate was adopted. The incidence, mortality, and population data for OC were subdivided into consecutive 5-year periods from 1990 to 2019. Individuals younger than 15 years and older than 95 years were arbitrarily categorized into one age group respectively since the incidence and mortality cases of OC in those younger than 15 years old and older than 95 years old are sparse.

Recently, Liu et al. evaluated several methods, including the Bayesian age-period-cohort model, generalized additive model, smooth spline model, Joinpoint model, and Poisson regression, to predict the liver cancer incidence burden from 1990 to 2030, they found that BAPC model, which based on Bayesian age-period-cohort analysis with integrated nested Laplace approximations (INLA), performed superior to other models with relatively lower absolute percentage deviation (APD) [3, 4]. In addition, Nordpred is also a common method based on Bayesian generalized age-period-cohort analysis, integrated with the power5 and poisson age-period-cohort models to estimate prediction of cancer incidence and mortality [5, 6]. And Lee et al. estimated the performances between Nordpred, log-linear Poisson model, autoregressive quadratic model, state-space model, and Joinpoint model to project the mortality and incidence in 12 cancers from Canadian cancer mortality data, they found that Nordpred provided better performance than the other methods [7]. Recently, Li et al. predicted the incidence and mortality burden of esophageal cancer in China by Nordpred, and the similar predicted results were validated by BAPC [8]. Therefore, we finally selected the Bayesian age-period-cohort model to predict the incidence and mortality of OC according to the above-mentioned results. Epidemiological distributions between esophageal cancer and OC were heterogeneous, and different Bayesian age-period-cohort algorithms between Nordpred and BAPC model were applied. To evaluate the predictive performance of those two methods in OC, we subdivided the whole China OC cohort into training sets (data from 1990 to 2014) and testing sets (data from 2015 to 2019) to train and test the predictive models respectively. The APD parameter is computed as the followed formulas:

$$APD=\frac{\left({\beta }_{predicted}-{\alpha }_{observed}\right)}{{\alpha }_{observed}} \times 100,$$

which was used to examine the model performance. Where the ${\beta }_{predicted}$ denotes the predictive value and the ${\alpha }_{observed}$ denotes the observational value. The lower APD determined the better the model. We compared APD results between the BAPC and Nordpred which were illustrated in Supplementary Fig. 1S and the total number of cases between observed values and predicted values were presented in Supplementary Fig. 2S. Age group-specific observational and predictive cases were displayed in Supplementary Table 1S. As a result, the BAPC model illustrated a superior performance with a lower APD than Nordpred in both incidence and mortality prediction in the China OC cohort. Finally, we determined that the BAPC model was used to forecast the incidence and mortality rates and case numbers of OC from 2020 to 2049. The Bayesian age-period-cohort model assumes that the observed age- and period-specific new cases or deaths counts fit a *Poisson* distribution, and the mean of the age- and period-specific new cases and deaths counts then is regressed on the effects of age, period, and cohort, using the corresponding population as the offset to predict future incidence and mortality [6]. We summarized the estimated variance parameters of incidence and mortality prediction in the BAPC models, including *mean*, *standard deviation*, 2.5% *quantile*,* median*, and 97.5% *quantile*, which are presented in Supplementary Tables 2S and 3S respectively. Statistics were performed via R opensource software platform (v4.0.2, R core team). A *p*-value of less than 0.05 was considered statistically significant.

## Results

### Incidence and mortality trends of ovarian cancer in China

**Fig. 1:**
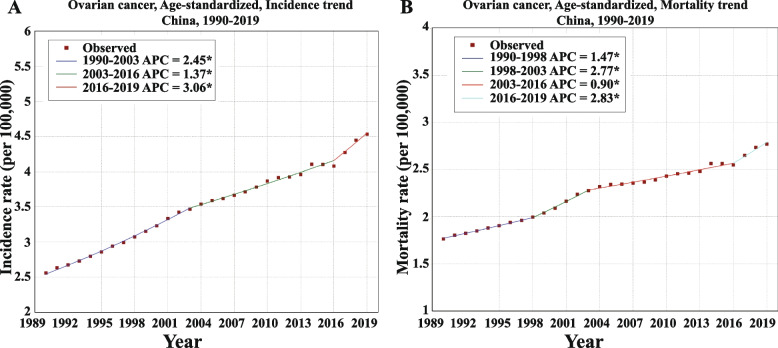
The age-standardized, temporal trends of incidence (**A**) and mortality (**B**) in ovarian cancer, China, 1990–2019. APC, annual percentage change; * *p* < 0.05

First, the overall age-standardized incidence rate (ASIR) increased by more than 2% (AAPC = 2.03; 95% CI, 1.90–2.16;* p* < 0.001) per year, and three consecutive temporal ASIR joinpoint trends between 1990 and 2019 were observed (Table 1 and Fig. 1A). The most notable annual increase period was from 2016 to 2019 (APC = 3.06; 95% CI, 1.88–4.24;* p* < 0.001) (Table 1). Second, like ASIR, the overall age-standardized mortality rate (ASMR) for OC in the Chinese female cohort significantly increased by 1.58% (AAPC = 1.58; 95% CI, 1.38 − 1.78; *p* < 0.001) per year from 1990 to 2019 and four consecutive temporal ASMR joinpoint trends between 1990 and 2019 were noted (Table 1 and Fig. 1B). Intriguingly, the most predominant annual increasing period in ASMR (from 2016 to 2019, APC = 2.83; 95% CI, 1.49–4.18;* p* < 0.001) was identical to ASIR (from 2016 to 2019, APC = 3.06; 95% CI, 1.88–4.24;* p* < 0.001) (Table 1).

**Table 1:** The APCs and AAPCs of incidence and mortality in ovarian cancer in China, 1990–2019

**Characteristics**	**Trend**	**Year**		**APC (%)**	**AAPC (%)**	**95% CI**		**Test Statistic**	***P*-value**
		**Start**	**End**			**Lower**	**Upper**		
**Age-standardized incidence rate**	1	1990	2003	2.45		2.33	2.58	42.43	< 0.001
	2	2003	2016	1.37		1.27	1.47	29.12	< 0.001
	3	2016	2019	3.06		1.88	4.24	5.45	< 0.001
		1990	2019		2.03	1.90	2.16	30.41	< 0.001
**Age-standardized mortality rate**	1	1990	1998	1.47		1.12	1.82	8.85	< 0.001
	2	1998	2003	2.77		2.04	3.52	7.94	< 0.001
	3	2003	2016	0.90		0.79	1.01	17.12	< 0.001
	4	2016	2019	2.83		1.49	4.18	4.46	< 0.001
		1990	2019		1.58	1.38	1.78	15.41	< 0.001

*APC* Annual percentage change, *AAPC* Average annual percentage change

### Predictions of ovarian cancer incidence and mortality burden in China

**Fig. 2:**
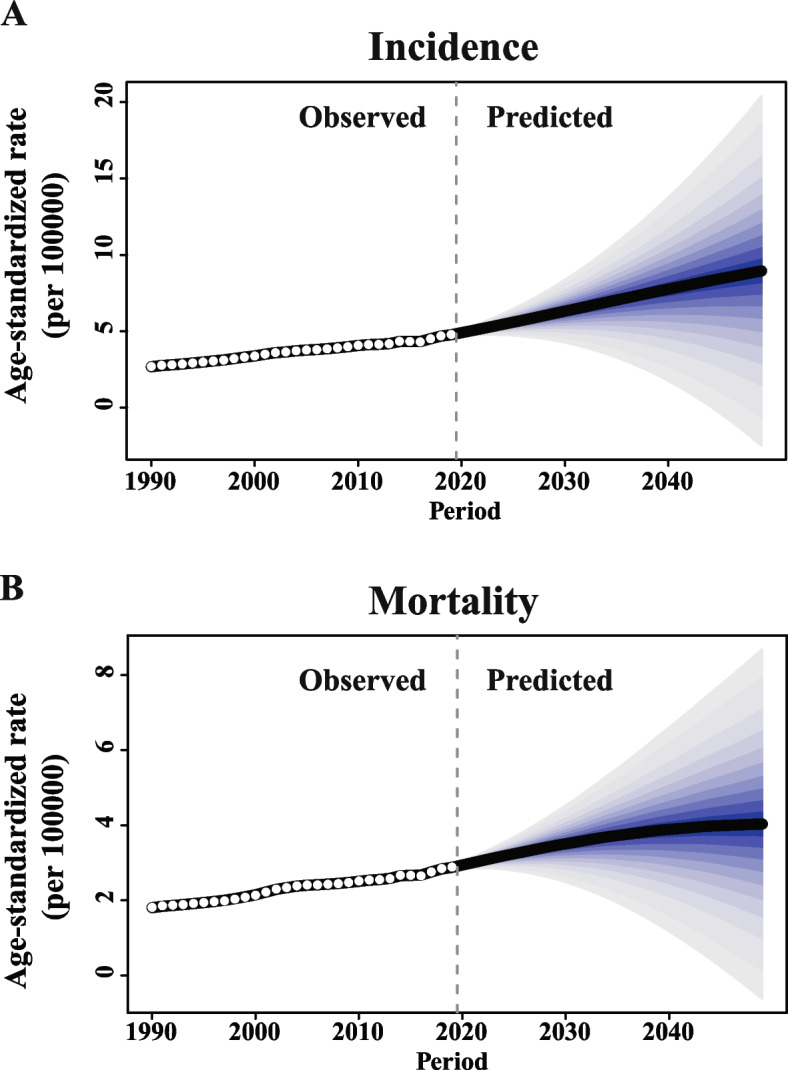
The trends of ASIR (**A**) and ASMR (**B**) in incidence and mortality in ovarian cancer, China, from 1990 to 2049. ASIR, age-standardized incidence rate (per 100,000 women), ASMR, age-standardized mortality rate (per 100,000 women). (Blue shading denotes 95% credible interval of estimates)

**Fig. 3:**
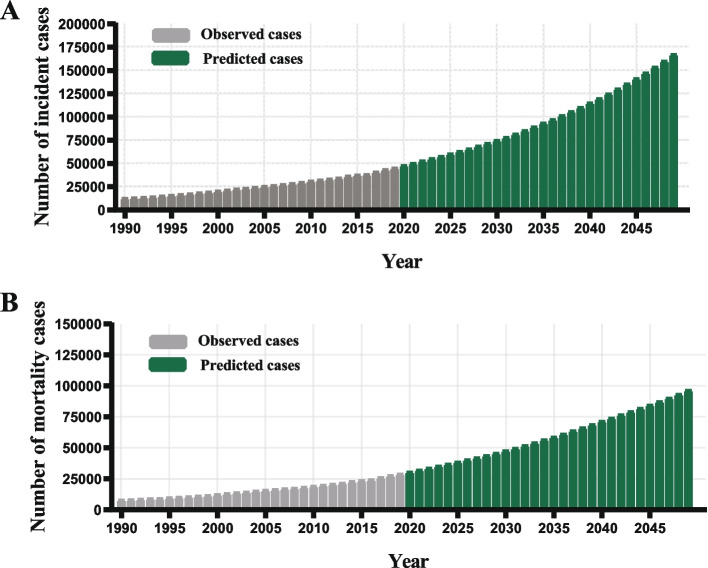
The number of new cases in incidence (**A**) and mortality (**B**) in ovarian cancer, China, from 1990 to 2049

Theoretically, both the estimated ASIR and ASMR of OC in the next 30 years in China estimated by Bayesian age-period-cohort analysis would continue to increase substantially, as well as the number of new cases in incidence and mortality (Figs. 2A, B and 3A, B). To be specific, the predicted ASIR would notably increase from 4.77 per 100,000 women in 2019 to 8.95 per 100,000 women in 2049 (Fig. 2A). Similarly, the predicted ASMR would consecutively increase from 2.88 per 100,000 women in 2019 to 4.03 per 100,000 women in 2049 (Fig. 2B). Consequently, the forecasted number of new cases would increase from 45,482 to 167,581 during the same period, and the forecasted number of deaths would increase from 29,092 to 96,501 (Fig. 3A and B).

## Discussion

With the estimated results in our study, the disease burden of incidence and mortality in OC in China significantly and consecutively increased from 1990 to 2019. Furthermore, these trends would continue to increase for the next 30 years based on the Bayesian age–period–cohort model prediction, which may bring a tremendous challenge for disease prevention and control in China in near future. According to the cancer statistics, ovarian cancer was the second leading cause of death with more than 27,000 estimated cancer deaths and 2.45 per 100,000 women compared to cervical (37,200 estimated cancer deaths, 3.36 per 100,000 women) and uterine (17,100 estimated cancer deaths, 1.51 per 100,000 women) cancer in 2016 in China [1]. Like our study, Huang et al. investigated age-standardized incidence and mortality rates from Shanghai Cancer Registry and found that both the incidence and mortality of OC increased in urban Shanghai from 1973 to 2012, with ASIR from 4.1 to 6.3 per 100,000 women and ASMR from 2.7 to 2.9 per 100,000 women respectively [9].

Although the specific etiologies of OC are unclear, no specific and sensitive screening and prevention methods are available and recommendatory for ovarian cancer heretofore [10, 11]. The increasing trends in OC incidence may be explained by some reasons. Evidence has indicated that breastfeeding was associated with the reduction of OC risk, however, low breastfeeding prevalence in China may have contributed to increasing the upward OC incidence trends [12–15]. A national representative survey with more than 14,000 children aged under 2 years from 55 counties in 30 provinces in China conducted in 2013 indicated that the crude exclusive breastfeeding rate under 6 months was only 20.7%, unsatisfactorily, and the weighted exclusive breastfeeding rate was less than 20% [13]. Similarly, another population-based, cross-sectional survey with more than 10,000 participants in 12 regions of China revealed that the exclusive breastfeeding rate was low (29.32% in 2017) and decreasing (from 32.71% in 2015 to 15.83% in 2017) among children aged 0–5 months [14].

Evidence had also demonstrated that overweight/obesity is associated with an increased risk of OC [16, 17]. The increasing prevalence of overweight/obesity in China may have also contributed to the rising incidence trend of OC [18]. The Chinese national prevalence estimates for 2015–19 were almost 35% for overweight and more than 16% for obesity in adults (≥ 18 years for both sexes), and the prevalence of obesity in women was more than 8% in 2004, and increasingly more than 14% in 2013–14 [18]. Likewise, data from the China Chronic Disease and Risk Factors Surveillance programme showed that obese women (37 million, 95% CI, 31 million—43 million) aged 18–69 years in China in 2018 were three times as many as in 2004 [19]. Other contributing factors, including nulliparity, lack of habitual physical activity, smoking, alcohol abuse, and absence of oral contraceptive use, may have been inordinately correlated with the rising trend of incidence of OC [20–23].

Accompanied by the increasing incidence estimates, the mortality of OC in China similarly presented an upward trend over the study period and will continue to rise for the next three decades owing to lack of availability of early diagnostic techniques, advanced stage at diagnosis, high recurrent rate, and resistance to chemotherapy [10, 20]. With the advancement of cancer genomic study, recent advanced research in poly (ADP-ribose) polymerase (PARP) inhibitor maintenance therapy for advanced epithelial serous ovarian carcinomas patients with BRCA1/2 mutation after finishing postoperative adjuvant first-line chemotherapy significantly prolonged the progress-free survival but the overall survival impact still needs to be investigated [24].

Inevitably, we must also acknowledge some limitations when interpreting the results collected from the GBD resources. Firstly, OC in GBD database composes of not only epithelial subtype but also the sex cord-stromal and germ cell tumor phenotypes. Besides, there are variances in incidence and mortality in urban and rural regions in China, but the specific data in urban and rural regions were not available. Additionally, data from the National Cancer Center of China contributed to GBD resources [1], only 27.60% of the national population was covered by the cancer registry data in 2016 which might thus not have fully presented the overall ovarian cancer disease burden. Finally, our predicted results were theoretically based on the Bayesian age–period–cohort model, the possibility of overestimation or underestimation to fit may not be avoided.

In the present study, we estimated the increasing disease burden of OC in incidence and mortality in China from 1990 to 2019 and projected the disease burden trends to 2049 via the Bayesian age–period–cohort model, which may facilitate the governors to establish relevant health policies that can guide practices to reduce the burden of ovarian cancer in China.

In conclusion, there were great challenges to prevent and control the disease burden of OC in China with the upward trends in incidence and mortality over the past 30 years. And the disease burden trends in incidence and mortality of OC will still aggravate for the coming three decades which indicates comprehensive measures to establish for OC prevention and treatment.

## Supplementary Information

**Additional file 1: Supplementary Table 1S.** Observational and predictive cases by age in incidence and mortality, 2015–2019.

**Additional file 2: Supplementary Table 2S.** Estimated variance parameters of incidence for ovarian cancer in BAPC models. The BAPC models assumes that the observed age- and period-specific new cases fit a Poisson distribution, and the mean of the age- and period-specific new cases then is regressed on the effects of age, period, and cohort, using the corresponding population as the offset to predict future incidence. The *mean, standard deviation, 2.5% quantile, median, and 97.5% quantile* by age categories in incidence of ovarian cancer are estimated.

**Additional file 3: Supplementary Table 3S.** Estimated variance parameters of mortality for ovarian cancer in BAPC models. The BAPC models assumes that the observed age- and period-specific deaths counts fit a Poisson distribution, and the mean of the age- and period-specific deaths counts then is regressed on the effects of age, period, and cohort, using the corresponding population as the offset to predict future mortality. The *mean, standard deviation, 2.5% quantile, median, and 97.5% quantile *by age categories in mortality of ovarian cancer are estimated.

**Additional file 4: Supplementary Figure 1S.** The absolute percentage deviation between the BAPC and Nordpred in incidence and mortality estimation respectively.

**Additional file 5: Supplementary Figure 2S.** The total number of cases between observed and predicted values. Predicted values were estimated by the BAPC (pink) and Nordpred (brown) package respectively.

## Data Availability

The datasets in this study are accessible in the GBD query tool at http://ghdx.healthdata.org/gbd-results-tool.

## Abbreviations

OC: Ovarian cancer
GBD: Global Burden of Disease
APC: Annual percent change
AAPC: Average annual percent change
APD: Absolute percentage deviation
CI: Confidence interval
ASIR: Age-standardized incidence rate
ASMR: Age-standardized mortality rate

## References

[1] Zheng R, Zhang S, Zeng H, Wang S, Sun K, Chen R (2022). Cancer incidence and mortality in China, 2016. J Natl Cancer Center.

[2] Wang Z, Guo E, Yang B, Xiao R, Lu F, You L (2021). Trends and age-period-cohort effects on mortality of the three major gynecologic cancers in China from 1990 to 2019: cervical, ovarian and uterine cancer. Gynecol Oncol.

[3] Liu Z, Xu K, Jiang Y, Cai N, Fan J, Mao X (2021). Global trend of aetiology-based primary liver cancer incidence from 1990 to 2030: a modelling study. Int J Epidemiol.

[4] Riebler A, Held L (2017). Projecting the future burden of cancer: Bayesian age-period-cohort analysis with integrated nested Laplace approximations. Biom J.

[5] Møller B, Fekjær H, Hakulinen T, Sigvaldason H, Storm HH, Talbäck M (2003). Prediction of cancer incidence in the Nordic countries: empirical comparison of different approaches. Stat Med.

[6] Jürgens V, Ess S, Cerny T, Vounatsou P (2014). A Bayesian generalized age-period-cohort power model for cancer projections. Stat Med.

[7] Lee TCK, Dean CB, Semenciw R (2011). Short-term cancer mortality projections: a comparative study of prediction methods. Stat Med.

[8] Li S, Chen H, Man J, Zhang T, Yin X, He Q (2021). Changing trends in the disease burden of esophageal cancer in China from 1990 to 2017 and its predicted level in 25 years. Cancer Med.

[9] Huang Z, Zheng Y, Wen W, Wu C, Bao P, Wang C (2016). Incidence and mortality of gynaecological cancers: secular trends in urban Shanghai, China over 40 years. Eur J Cancer.

[10] Menon U, Karpinskyj C, Gentry-Maharaj A (2018). Ovarian cancer prevention and screening. Obstet Gynecol.

[11] Henderson JT, Webber EM, Sawaya GF (2018). Screening for ovarian cancer: updated evidence report and systematic review for the US Preventive Services Task Force. JAMA.

[12] Li DP, Du C, Zhang ZM, Li GX, Yu ZF, Wang X (2014). Breastfeeding and ovarian cancer risk: a systematic review and meta-analysis of 40 epidemiological studies. Asian Pac J Cancer Prev.

[13] Duan Y, Yang Z, Lai J, Yu D, Chang S, Pang X (2018). Exclusive breastfeeding rate and complementary feeding indicators in China: a national representative survey in 2013. Nutrients.

[14] Fang Z, Liu Y, Wang H, Tang K (2020). The patterns and social determinants of breastfeeding in 12 selected regions in China: a population-based cross-sectional study. J Hum Lact.

[15] Luan NN, Wu QJ, Gong TT, Vogtmann E, Wang YL, Lin B (2013). Breastfeeding and ovarian cancer risk: a meta-analysis of epidemiologic studies. Am J Clin Nutr.

[16] Dixon SC, Nagle CM, Thrift AP, Pharoah PDP, Pearce CL, Zheng W (2016). Adult body mass index and risk of ovarian cancer by subtype: a Mendelian randomization study. Int J Epidemiol.

[17] Olsen CM, Green AC, Whiteman DC, Sadeghi S, Kolahdooz F, Webb PM (2007). Obesity and the risk of epithelial ovarian cancer: a systematic review and meta-analysis. Eur J Cancer.

[18] Pan XF, Wang L, Pan A (2021). Epidemiology and determinants of obesity in China. Lancet Diabetes Endocrinol.

[19] Wang L, Zhou B, Zhao Z, Yang L, Zhang M, Jiang Y (2021). Body-mass index and obesity in urban and rural China: findings from consecutive nationally representative surveys during 2004–18. Lancet.

[20] Matulonis UA, Sood AK, Fallowfield L, Howitt BE, Sehouli J, Karlan BY (2016). Ovarian cancer. Nat Rev Dis Primers.

[21] Hunn J, Rodriguez GC (2012). Ovarian cancer: etiology, risk factors, and epidemiology. Clin Obstet Gynecol.

[22] Reid BM, Permuth JB, Sellers TA (2017). Epidemiology of ovarian cancer: a review. Cancer Biol Med.

[23] Momenimovahed Z, Tiznobaik A, Taheri S, Salehiniya H (2019). Ovarian cancer in the world: epidemiology and risk factors. Int J Womens Health.

[24] Tew WP, Lacchetti C, Ellis A, Maxian K, Banerjee S, Bookman M (2020). PARP inhibitors in the management of ovarian cancer: ASCO guideline. J Clin Oncol.

