# Supplementary material for: Trends in incidence and mortality for ovarian cancer in China from 1990 to 2019 and its forecasted levels in 30 years
Source: J Ovarian Res. 2023 Jul 14;16:139. doi: 10.1186/s13048-023-01233-y (PMC10347789; doi:10.1186/s13048-023-01233-y)
Supplement: Supplementary file 1 — Additional file 1: Supplementary Table 1S. Observational and predictive cases by age in incidence and mortality, 2015–2019. [file 13048_2023_1233_MOESM1_ESM.pdf]

Supplementary Table 1S. Age group-specific observational and predictive cases in incidence and mortality, 2015 - 2019.

	Incidence			Mortality		
Age group	Observed	Nordpred	BAPC	Observed	Nordpred	BAPC
0-14	178	199	171	30	34	27
15-19	309	294	294	44	40	40
20-24	660	538	560	102	79	83
25-29	1001	939	933	170	155	151
30-34	1228	1246	1267	288	284	278
35-39	1621	1463	1508	441	380	386
40-44	2874	2700	2755	1002	905	916
45-49	4551	4635	4808	1972	1918	1979
50-54	5999	5788	5799	3398	3091	3143
55-59	5047	4906	5150	3247	3008	3147
60-64	5449	5502	5497	3989	3905	3963
65-69	4888	4925	5024	3985	3931	4052
70-74	3408	3323	3324	3150	3033	3088
75-79	2024	2059	2090	2113	2129	2181
80-84	1311	1363	1370	1373	1432	1466
85-89	526	599	617	677	761	786
90-94	179	176	175	245	241	246
95+	29	27	28	48	43	46
