# Supplementary figures and images for: Trends in incidence and mortality for ovarian cancer in China from 1990 to 2019 and its forecasted levels in 30 years
Source: J Ovarian Res. 2023 Jul 14;16:139. doi: 10.1186/s13048-023-01233-y (PMC10347789; doi:10.1186/s13048-023-01233-y)

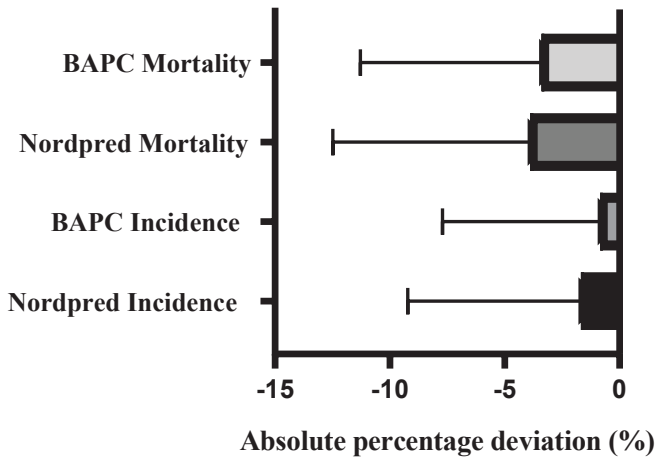

Supplement: Supplementary file 4 — Additional file 4: Supplementary Figure 1S. The absolute percentage deviation between the BAPC and Nordpred in incidence and mortality estimation respectively. [file 13048_2023_1233_MOESM4_ESM.pdf]

**Number of cases  
2015 - 2019**

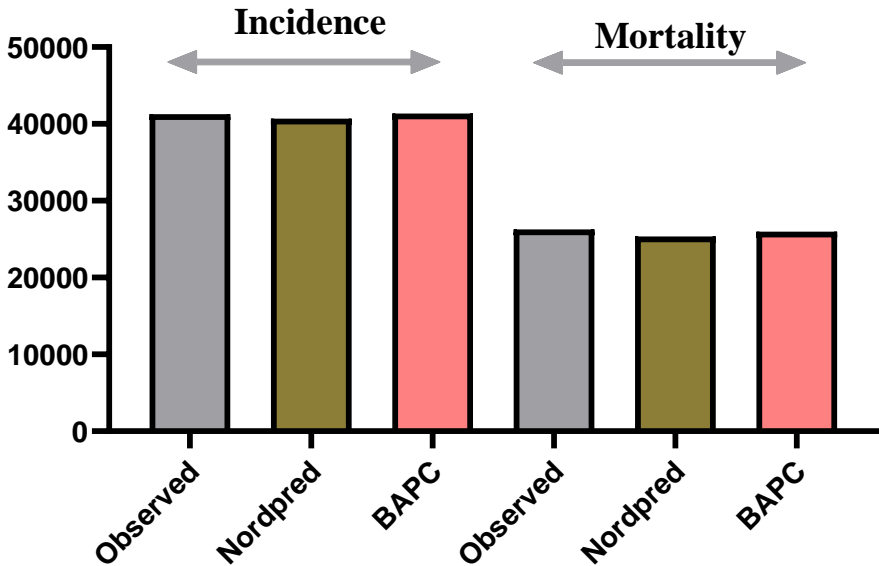

Supplement: Supplementary file 5 — Additional file 5: Supplementary Figure 2S. The total number of cases between observed and predicted values. Predicted values were estimated by the BAPC (pink) and Nordpred (brown) package respectively. [file 13048_2023_1233_MOESM5_ESM.pdf]
